# Unraveling the Role of α2δ‐1 in Cerebral Hemorrhage: Calcium Overload, Endoplasmic Reticulum Stress, and Microglial Apoptosis

**DOI:** 10.1002/brb3.70499

**Published:** 2025-05-07

**Authors:** Ning Yu, Xiaopeng Li, Bingqian Wang, Chengrui Nan, Qianxu Jin, Liang Yang, Depei Li, Zongmao Zhao

**Affiliations:** ^1^ Department of Anesthesiology and intensive care unit The Second Hospital of Hebei Medical University Shijiazhuang Hebei Province China; ^2^ Department of Neurosurgery The First Hospital of Handan City Handan Hebei Province China; ^3^ Department of Neurosurgery Affiliated Xing Tai People Hospital of Hebei Medical University Xingtai Hebei Province China; ^4^ Department of Neurosurgery The Second Hospital of Hebei Medical University Shijiazhuang Hebei Province China; ^5^ Department of Medicine University of Missouri School of Medicine One Hospital Drive Columbia Missouri USA

**Keywords:** calcium signaling, cerebral hemorrhage endoplasmic reticulum stress, α2δ‐1

## Abstract

**Objective:**

Cerebral hemorrhage is a severe condition associated with high morbidity and mortality. Understanding the underlying pathogenesis is crucial for developing effective therapeutic strategies. This study aimed to investigate the role of the dysregulated α2δ‐1 protein in cerebral hemorrhage.

**Materials and Methods:**

We observed a significant upregulation of α2δ‐1 in cerebral hemorrhage tissue. Knockdown of α2δ‐1 resulted in decreased intracellular calcium concentration and reduced phosphorylation of PLCr and IP3R in the presence of calcium. Additionally, α2δ‐1‐mediated calcium overload induced ERS in BV2 microglia, accompanied with increased phosphorylation of PERK and decreased ERS‐related protein levels.

**Results:**

α2δ‐1 knockdown significantly inhibited BV2 microglia apoptosis and downregulated apoptosis‐related proteins in the presence of calcium. Our study indicates the involvement of α2δ‐1 in calcium‐mediated signaling, endoplasmic reticulum stress, and BV2 microglia apoptosis.

**Conclusions:**

The findings provide a basis for considering α2δ‐1 as a potential therapeutic target in cerebral hemorrhage and secondary brain injury conditions associated with calcium dysregulation.

## Graphical abstract

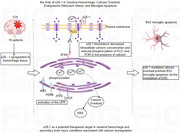

## 1 Introduction

Intracerebral hemorrhage (ICH) is the second most common type of stroke, accounting for approximately 10% to 20% of all strokes. ICH leads to higher mortality and more severe disability compared to ischemic stroke, with the higher global burden of disease and the most serious public health problems worldwide (An et al. 2017). It is caused by the rupture of cerebral blood vessels and subsequent leakage of blood, including intrinsic factors, into the brain parenchyma (Ren et al. 2020). Intracerebral hemorrhage not only results in direct compression and occupying injuries caused by the hematoma but also leads to secondary brain injury (SBI), which significantly worsens the condition of ICH patients. The progression of perihematomal edema (PHE) following intracerebral hemorrhage is recognized as a critical factor in secondary brain injury, which is associated with various processes, such as thromboxane‐induced blood‐brain barrier disruption, brain edema, neuronal death, release of excitotoxic substances from red blood cell lysis, oxidative stress, ferroptosis and neurotoxicity, activation of microglia and macrophages, and initiation of inflammatory response pathways (Ren et al. 2020, Panther et al. 2022, Chen et al. 2021). Microglia can be activated through hematoma‐derived blood components, inflammatory mediators, oxidative stress responses, and autophagy mechanisms to maintain homeostasis after ICH. This activation process facilitates the secretion of pro‐inflammatory factors, anti‐inflammatory factors, inflammatory chemokines, and oxidative stress‐associated mediators (Hanisch and Kettenmann 2007). Secondary brain injury plays a crucial role in neurocyte damage following cerebral hemorrhage (Wilkinson et al. 2018). Despite ongoing advancements in medical science, effective treatment methods for cerebral hemorrhage remain elusive (An et al. 2017). Consequently, there is an urgent need to delve into the underlying mechanisms involved, as this exploration holds tremendous potential as a novel and promising strategy for enhancing the treatment of ICH.

Calcium ion (Ca^++^) is an essential second messenger involved in the regulation of multiple intracellular signaling pathways and mediates various physiological functions. Voltage‐gated ion channels (VGCC) are transmembrane protein complexes that modulate ion channel activity by altering the membrane potential near the channels (Pchitskaya et al. 2018, Nguyen et al. 2020). Calcium influx plays a critical role in the physiological and pathophysiological processes of numerous neurological diseases (Wang et al. 2017). α2δ‐1 is an auxiliary subunit of voltage‐gated calcium channels. The function and assembly of voltage‐gated calcium channels rely on the association of the pore‐forming subunit α1 with the auxiliary subunits β and α2δ. Research has indicated that the α2δ‐1‐NMDAR complex in the hypothalamus serves as a crucial molecular substrate for the interaction between the sympathetic nervous system and the renin‐angiotensin system, suggesting that α2δ‐1 could be a potential target for treating neurogenic hypertension (Ma et al. 2018). The transport of the pore‐forming subunit α1 to the plasma membrane requires the β subunit, and the α2δ subunit further enhances forward transport and promotes surface expression of the channel. The α2δ family has a weak effect on VGCC alone and requires the synergistic action of β subunits to upregulate calcium currents by facilitating the folding of pore‐forming channels (i.e., maturation of VGCC) and preventing their degradation by the endoplasmic reticulum (Dolphin 2016, Cassidy et al. 2014, Dolphin 2012, Martínez San Segundo et al. 2020).

Endoplasmic reticulum stress (ERS) refers to the activation of unfolded protein response, endoplasmic reticulum overload response, and caspase‐12 by cells in response to the aggregation of misfolded and unfolded proteins in the endoplasmic reticulum lumen and dysregulation of calcium homeostasis, leading to apoptosis mediated by apoptosis pathways and other signaling pathways (Schönthal 2009). The endoplasmic reticulum transmits appropriate calcium signals to the mitochondria, which then decode them into specific inputs to regulate essential functions such as metabolism, energy production, and apoptosis (Marchi et al. 2018). A study investigating the mechanisms of allicin in mitigating myocardial ischemia‐reperfusion (MI/R) injury demonstrated that its protective effect might be associated with the inhibition of Ca2+ overload‐induced apoptosis and the inhibition of the PI3K ‐mediated GRK2/PLC‐γ/IP3R signaling pathway (Gao et al. 2021).

In our study, we aim to investigate the expression of α2δ‐1 in the brain tissue surrounding the hematoma after ICH and elucidate the role of α2δ‐1 in cerebral hemorrhage. Through exploring the molecular mechanism in BV2 microglia cell, we have verified that PLC‐γ phosphorylation activation can accelerate the apoptosis of cerebral hemorrhage cells through α2δ‐1 Ca^2+^ signaling pathway.

## 2 Materials and Methods

### 2.1 Patient and Sample Collection

This study was conducted in accordance with the Helsinki Declaration and was approved by the Research Ethics Committee of the Second Hospital of Hebei Medical University (Approval letter NO.2017‐R232). We conducted a study involving 16 patients (mean age: 57.38 ± 10.36 years, range: 37–77 years) with spontaneous basal ganglia hemorrhage who underwent surgery within 24 h, as detailed in Table 1. Prior to surgical resection, these patients provided written informed consent and did not receive any treatment. Cerebral hemorrhage tissue and peripheral tissue samples were collected for analysis. Samples were collected at a standard distance of 1 cm away from the edge of the hematoma and labeled as peripheral brain tissue. Brain tissue near the edge of the hematoma was labeled as cerebral hemorrhage tissue. And the minimum volume of each sample was 0.5 cm^3^. The collection procedure was performed by the same surgeon, utilizing a cortical fistula created based on the location of the cerebral hemorrhage. The distance between the specimen and the hematoma was directly measured. Following collection, the brain tissue was promptly frozen in liquid nitrogen or fixed in formalin for preservation.

**TABLE 1:** Demographic characteristics of patients with spontaneous basal ganglia hemorrhage.

Name	Id	Sex	Age(years)	ICH(ml)	Position	GCS(score)
Wang Jinxiang	2110777	2	56	85	1	9
Jia Fuzhen	2141425	2	70	45	1	8
Ding Shucong	2197165	2	54	40	1	10
Meng Xishun	2202924	1	73	35	2	12
Xu Xuebing	2221525	1	48	43	2	10
Zhao Zhilan	2259490	1	64	60	1	8
Yuan Changqi	2138366	1	59	35	2	10
Lv Jinzhi	2138733	2	57	30	2	12
Qin Zhaoyi	2233739	1	47	73	2	6
Wang Jianli	2235311	1	49	50	1	9
Li Jianxue	2114222	1	54	55	1	10
Wang Wenxue	2238935	1	62	60	1	8
Feng Shulian	2239541	2	51	70	2	7
Wang Xiaoling	2239308	2	77	68	1	6
Liu Xianqi	2167791	1	37	82	1	7
Gao Shuangxia	2174452	2	60	37	1	11

Sex: 1 = male, 2 = female.

POSITION: 1 = left, 2 = right.

### 2.2 Cell Culture

BV2, was obtained from the American Type Culture Collection and cultured in complete medium consisting of MEM medium (11095080, thermo fisher scientific) supplemented with 2 mM glutamine, 100 U/ml penicillin, 100 mg/ml streptomycin, and 10% fetal bovine serum (10099141, thermo fisher scientific) and maintained at 37°C under 5% CO_2_.

### 2.3 α2δ1 Gene Knockdown Assay

Cells at approximately 70–80% confluency were transfected using Lipofectamine 3000 (L3000008, thermo). The α2δ1 gene was cloned into the PLKO.1‐TRC‐puro vector for the experimental group, while the control group received the empty PLKO.1‐TRC‐puro vector. 2 µg of the plasmid DNA was diluted in 50 µL of Opti‐MEM medium (51985091, thermo fisher Scientific). Simultaneously, 5 µL of P3000 reagent was added to the DNA solution and mixed gently. In a separate tube, 3 µL of lipofectamine 3000 was diluted in 50 µL of Opti‐MEM medium. The diluted DNA was combined with the diluted lipofectamine 3000, mixed gently, and incubated for 15 min at room temperature to form transfection complexes. The cell culture medium was replaced with fresh DMEM without antibiotics, and the transfection complexes were added dropwise to the cells, which were then gently mixed. The cells were incubated at 37°C in a 5% CO_2_ humidified incubator for 6 h. After 6 h, the medium was replaced with fresh DMEM containing 10% FBS and antibiotics. Following 48 h post‐transfection, the cells were treated with puromycin (2 µg/mL) to select for successfully transfected cells. The cells were maintained under puromycin selection for 5–7 days until colonies formed. Single colonies were picked and expanded for further analysis.

### 2.4 Immunofluorescence Staining of Hematomal Tissues

Tissue samples from peripheral and cerebral hemorrhage regions of human brain specimens were fixed in 4% paraformaldehyde (P0099, Beyotime) for 24 h, then washed with running water. Dehydration was performed using a graded ethanol series (50%, 70%, 85%, 95%, and 100%) and cleared in xylene. Tissues were embedded in paraffin, sectioned at 5 µm, and mounted on poly‐L‐lysine‐coated slides. Sections were deparaffinized in xylene, rehydrated through graded ethanol, and washed in PBS (AR0030, Boster). Endogenous peroxidase was blocked with 3% H_2_O_2_ for 10 min, and antigen retrieval was performed in sodium citrate buffer (pH 6.0) at 95°C for 10 min. After blocking with normal goat serum, sections were incubated with α2δ1 primary antibody (DF8510, affinity) overnight at 4°C, followed by HRP conjugated goat anti‐human IgG for 90 min at room temperature. Sections were counterstained with DAPI, and stained sections were observed and photographed under a fluorescence microscope at 200× magnification (CKXX53, OLYMPUS).

### 2.5 Western Blot Assays

Western blotting was performed the expression of apoptosis‐related proteins in BV2 cells, ER stress‐related proteins in BV2 cells, α2δ1 protein in peripheral tissue of intracebral hemorrhage of human species and cerebral hemorrhage tissue of human species. Antibodies to α2δ‐1 (BE3455, EASYBIO) or Bax (ab32503, Abcam), cleaved caspase‐3 (ab2302, Abcam), cleaved caspase‐9 (ab2324, Abcam), cleaved caspase‐12 (ab62463, Abcam), Phospho‐PERK (3179, CST), CHOP (5554, CST), ATF6 (ab2576, Abcam), phospho‐IRE1(ab48187, Abcam) were from Abcam and EASYBIO. Tissues or BV2 cells were harvested and homogenized in RIPA buffer (Roo2, Solarbio). Proteins were separated by SDS–PAGE (5% stacking gel and 10% separated gel) (AR0138, BOSTER) and transferred to PVDF membrane (IPVH0010, Millipore). The membranes were blocked with 5% non‐fat dry milk in TBST buffer for 2 h at room temperature. The membranes were then incubated overnight at 4°C with the primary antibody. Afterwards, the membranes were incubated with the secondary antibody for 1.5 h at room temperature. The protein bands were visualized using Western Lightning^TM^ Chemiluminescence Reagent (NEL10300EA, PerkinElmer) and quantified using Epson Perfection V39 system (V39, EPSON).

### 2.6 Flow Cytometry Detection of Cell Apoptosis

We have established the following four groups for detecting cell apoptosis: NC+calcium medium+5%O_2_ hypoxic culture; α2δ1‐KD+calcium medium+5%O_2_ hypoxic culture; NC+calcium free medium+5%O_2_ hypoxic culture and α2δ1‐KD +calcium free medium+5%O_2_ hypoxic culture. 5×10^4^‐10^5^ BV2 cells were collected and followed staining according to the instructions provided in the Annexin V‐FITC kit (AP101‐100‐kit, LiankeBio). Perform the detection on BD FACSCaliburTM Flow Cytometer (E97501093, BD Biosciences).

### 2.7 Detect p‐PERK Expression by Immunofluorescence Assay

Cells were divided into four groups: α2δ1‐NC with calcium medium under 5% O2 hypoxic culture, α2δ1‐KD with calcium medium under 5% O2 hypoxic culture, α2δ1‐NC with calcium‐free medium under 5% O2 hypoxic culture, and α2δ1‐KD with calcium‐free medium under 5% O2 hypoxic culture. ER‐Tracker Green (C1042s, Beyotime) was diluted at a ratio of 1:1000 into the ER‐Tracker Green dilution solution, which was pre‐warmed at 37°C before use. The cell culture medium was removed, and the cells grown on coverslips were washed with washing solution. Then, the cells were incubated with the pre‐warmed ER‐Tracker Green working solution at 37°C for 30 min. The cells were then washed three times with PBS, incubated in 0.5% Triton X‐100 for 10 min, and blocked with 2% bovine serum albumin for 1 h at room temperature. Following this, the cells were incubated with the p‐PERK (ab65142, Abcam) at 4°C overnight and washed three times with PBS. The goat anti‐mouse IgG (PA5‐59067, Abcam) was applied for 2 h at room temperature before another PBS wash. After air drying, the cells were observed and photographed under a fluorescence microscope (CKXX53, OLYMPUS). Images were semi‐quantitatively analyzed using Image Pro Plus software to calculate the average fluorescence intensity.

### 2.8 Intracellular Calcium Ion Concentration Detection

BV2 cells were fixed with 4% formalin solution and then permeabilized with 0.1% Triton X‐100, followed by blocking with 5% bovine serum albumin solution. Then, cells were incubated with 0.5 µM fura‐2 AM probe (s1052, Beyotime) for 60 min at room temperature. The cells were washed twice with PBS (C3580‐0500, BioMed) and followed stained with Hoechst 33342 and fluorescence labeling of the cells was performed using Alexa Fluor 488‐conjugated anti‐IgG antibody (ab18447, Abcam). Finally, images of the cell were captured under a fluorescence microscope (CKXX53, OLYMPUS).

### 2.9 Statistical Analysis

In the experiment, all data are presented as the mean standard error of the three experimental means. Unpaired data was analyzed using Student's t‐test. Multiple sets of data were compared using one‐way analysis of variance (GraphPad version 7.0, GraphPad Software, CA, USA), and the Tukey's honest significant difference (HSD) test was utilized as the post hoc test following the ANOVA. The observed difference was found to be statistically significant at a significance level of *P* < 0.05.

## 3 Results

### 3.1 Elevated Levels of α2δ‐1 Protein in Cerebral Hemorrhage Tissue

**FIGURE 1:**
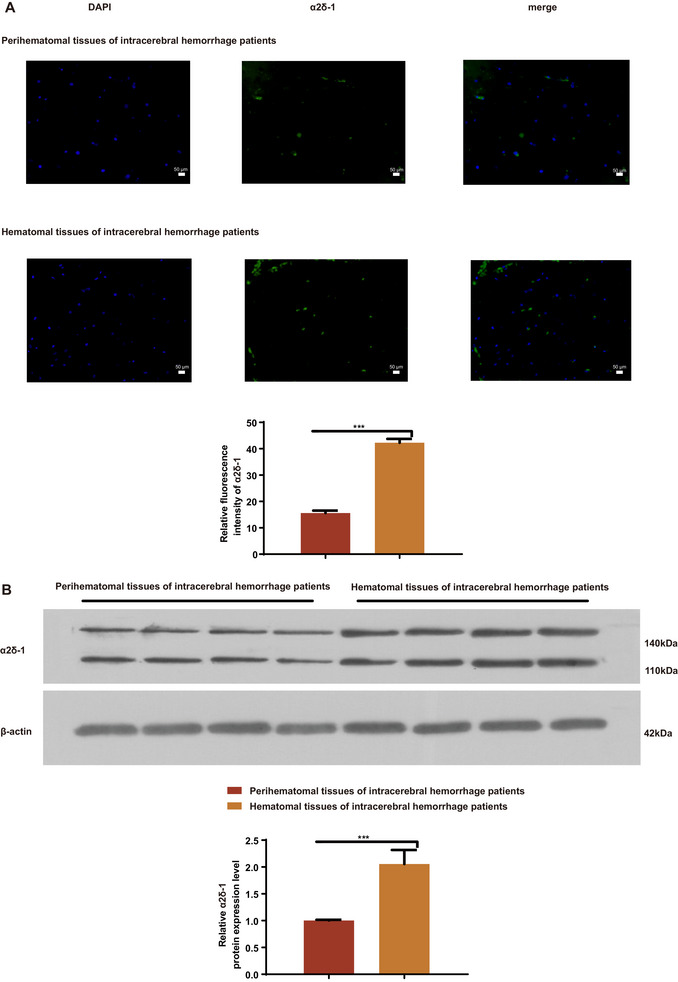
Elevated levels of α2δ‐1 protein in cerebral hemorrhage tissue compared to peripheral tissue in intracerebral hemorrhage patients. (A) Representative images of the localization of α2δ‐1 in cerebral hemorrhage tissue and peripheral tissue of intracerebral hemorrhage patients. Nuclei were stained with DAPI (blue), and α2δ‐1 was visualized in green (magnification: 40×; scale bar: 50 µm). The bar graph presents the relative fluorescence intensity of α2δ‐1 in cerebral hemorrhage tissue and peripheral tissue. (B) Western blot analysis of α2δ‐1 expression in human cerebral hemorrhage tissue and peripheral tissue of intracerebral hemorrhage patients. β‐actin was used as a loading control. Representative blots from two groups are shown. The results demonstrate a significant upregulation of α2δ‐1 in cerebral hemorrhage tissue compared to peripheral tissue. Data are presented as the mean ± standard error of the mean (SEM). *, *P* < 0.05, **, *P* < 0.01, ***, *P* < 0.001. Error bars represent the SEM. Statistical analysis was performed using an unpaired, two‐tailed student's t‐test.

In our study, we screened 27 patients, ultimately enrolling 16 cases, and collected cerebral hemorrhage tissue and peripheral tissue samples from patients with intracerebral hemorrhage (ICH) in the basal ganglia who underwent emergency surgery at the Second Hospital of Hebei Medical University. The expression of α2δ‐1 protein was assessed using immunofluorescence (Figure 1A) and Western Blot (Figure 1B). Our findings revealed a significant upregulation of α2δ‐1 in cerebral hemorrhage tissue compared to peripheral tissue at the protein expression level.

### 3.2 α2δ1‐Mediated Calcium Overload Promotes Phosphorylation of PLCγ and IP3R in BV2 Microglia

**FIGURE 2:**
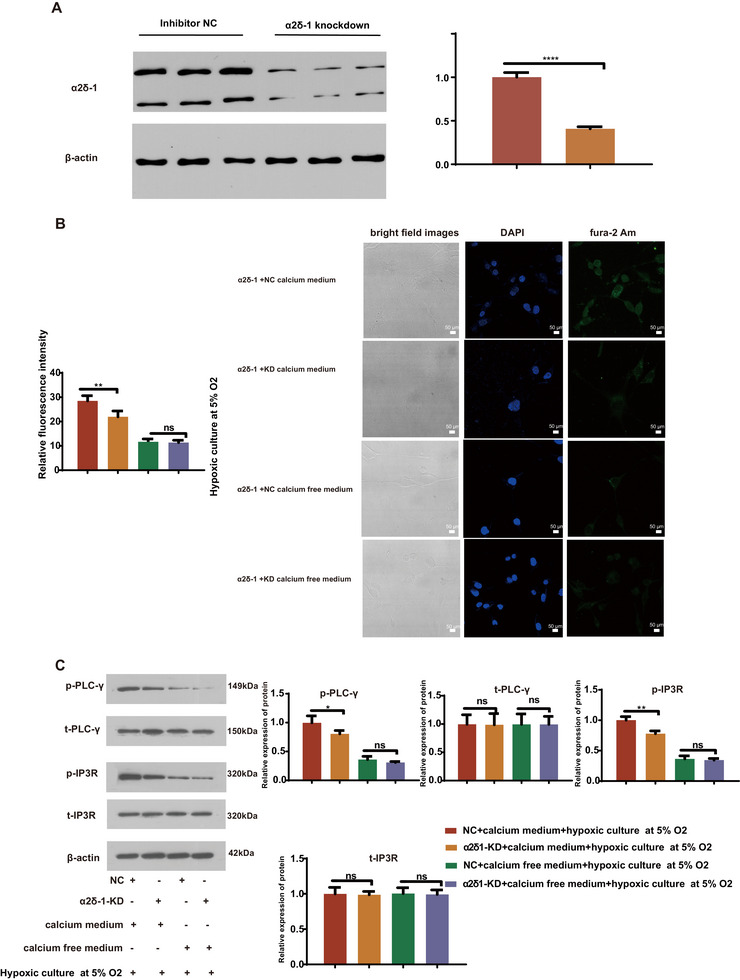
α2δ1‐mediated calcium overload promotes phosphorylation of PLCr and IP3R in BV2 microglia. (A) The relative protein expression level of α2δ‐1 was quantified after knockdown (KD). The efficacy of α2δ‐1 knockdown was confirmed using an inhibitor as a negative control (inhibitor NC), with β‐actin as a reference. (B) Representative images of BV2 microglia stained with multiplex immunofluorescence, highlighting the intracellular calcium levels (green) using fura‐2 AM staining and the nuclei (blue) using DAPI staining (magnification: 40×; scale bar: 50 µm). The bar graph presents the relative fluorescence intensity based on four independent experiments. The results demonstrate a significant reduction in intracellular calcium levels following α2δ1 knockdown, indicating that extracellular calcium enters BV2 microglia through α2δ1 calcium channels, leading to cytoplasmic calcium overload. (C) Western blot analysis of the phosphorylation of PLCr and IP3R in BV2 microglia. β‐actin was used as a loading control. Representative blots from four independent experiments are shown. The results reveal that intracellular calcium overload promotes the phosphorylation of PLCr and IP3R in BV2 microglia. Data are presented as the mean ± standard error of the mean (SEM). *, *P* < 0.05, **, *P* < 0.01, ***, *P* < 0.001. Error bars represent the SEM. Statistical analysis was performed using an unpaired, two‐tailed student's t‐test.

In order to investigate the impact of α2δ1 on intracellular and extracellular calcium ions, we conducted immunofluorescence staining of BV2 cells. The BV2 cells were subjected to 5% oxygen hypoxia and heme treatment, and were divided into calcium‐containing and calcium‐free culture conditions. The α2δ‐1 Knockdown (α2δ‐1 KD) and inhibitor NC were transfected into the cells. Western blot analysis revealed that the expression of α2δ‐1 protein was significantly downregulated in the α2δ‐1 KD group compared to the inhibitor NC group (Figure 2A). The intracellular calcium concentration was measured, and the results showed that the α2δ‐1 KD group exhibited a significant decrease in Ca2+ concentration compared to the inhibitor NC and control groups in the calcium culture, while no notable change was observed in the calcium ion‐free culture (Figure 2B). Western blot analysis was performed to assess the phosphorylation of PLC**γ** and IP3R. The findings revealed that the knockdown of α2δ‐1 significantly reduced the protein expression levels of phosphorylated PLC**γ** and IP3R in the calcium‐containing culture, whereas no significant changes were observed in the calcium‐free culture (Figure 2C).

### 3.3 α2δ‐1‐Mediated Calcium Overload Induces Endoplasmic Reticulum Stress Response in BV2 Microglia

**FIGURE 3:**
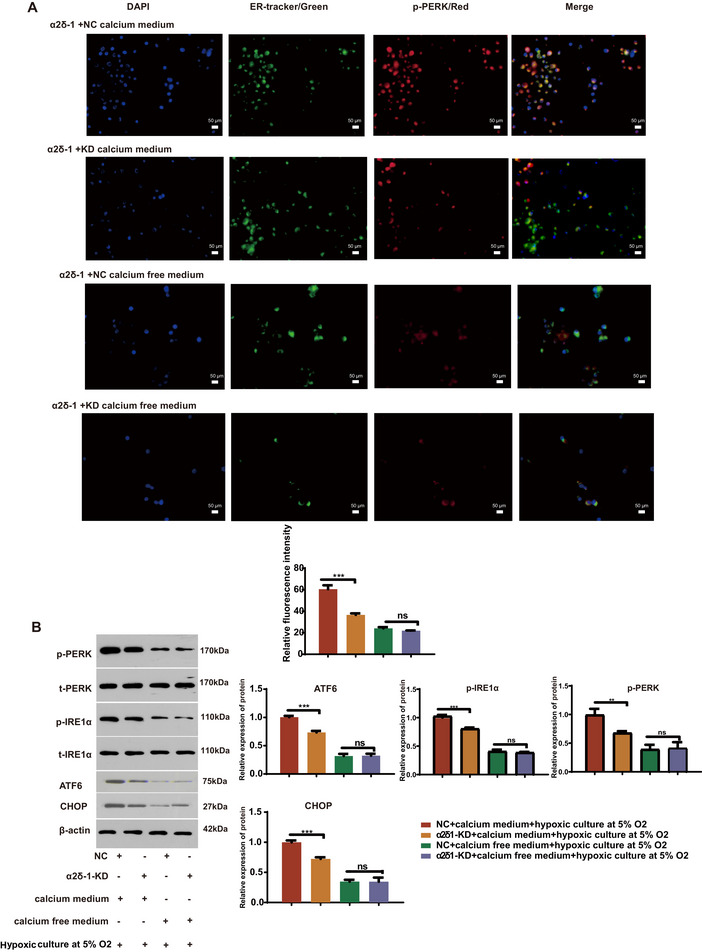
Intracellular calcium overload induces endoplasmic reticulum stress in BV2 microglia. (A) Representative images of BV2 microglia stained with multiplex immunofluorescence, highlighting the endoplasmic reticulums (green) and p‐PERK (red) (magnification: 40×; scale bar: 50 µm). The bar graph presents the relative fluorescence intensity based on four independent experiments. The results demonstrate that intracellular calcium overload promotes the phosphorylation of PERK in BV2 microglia. (B) Western blot analysis of endoplasmic reticulum stress‐related proteins in BV2 microglia. β‐actin was used as a loading control. Representative blots from four independent experiments are shown. The results reveal that intracellular calcium overload promotes the expression of p‐PERK, p‐IRE1α, ATF6, and CHOP in BV2 microglia. Data are presented as the mean ± standard error of the mean (SEM). *, *P* < 0.05, **, *P* < 0.01, ***, *P* < 0.001. Error bars represent the SEM. Statistical analysis was performed using an unpaired, two‐tailed student's t‐test.

In this study, BV2 cells were cultured in a simulated post‐cerebral hemorrhage environment to investigate the effect of calcium overload on the endoplasmic reticulum stress response. Immunofluorescence staining demonstrated increased phosphorylation of PERK (p‐PERK) in BV2 microglia, indicating activation of endoplasmic reticulum stress due to intracellular calcium overload (Figure 3A). Western blot analysis further confirmed that α2δ‐1 knockdown resulted in decreased expression of endoplasmic reticulum stress‐related proteins (CHOP, PERK, IRE1, and ATF6) specifically in the presence of calcium (Figure 3B).

### 3.4 α2δ‐1‐Mediated Calcium Overload Promoted BV2 Microglia Apoptosis

**FIGURE 4:**
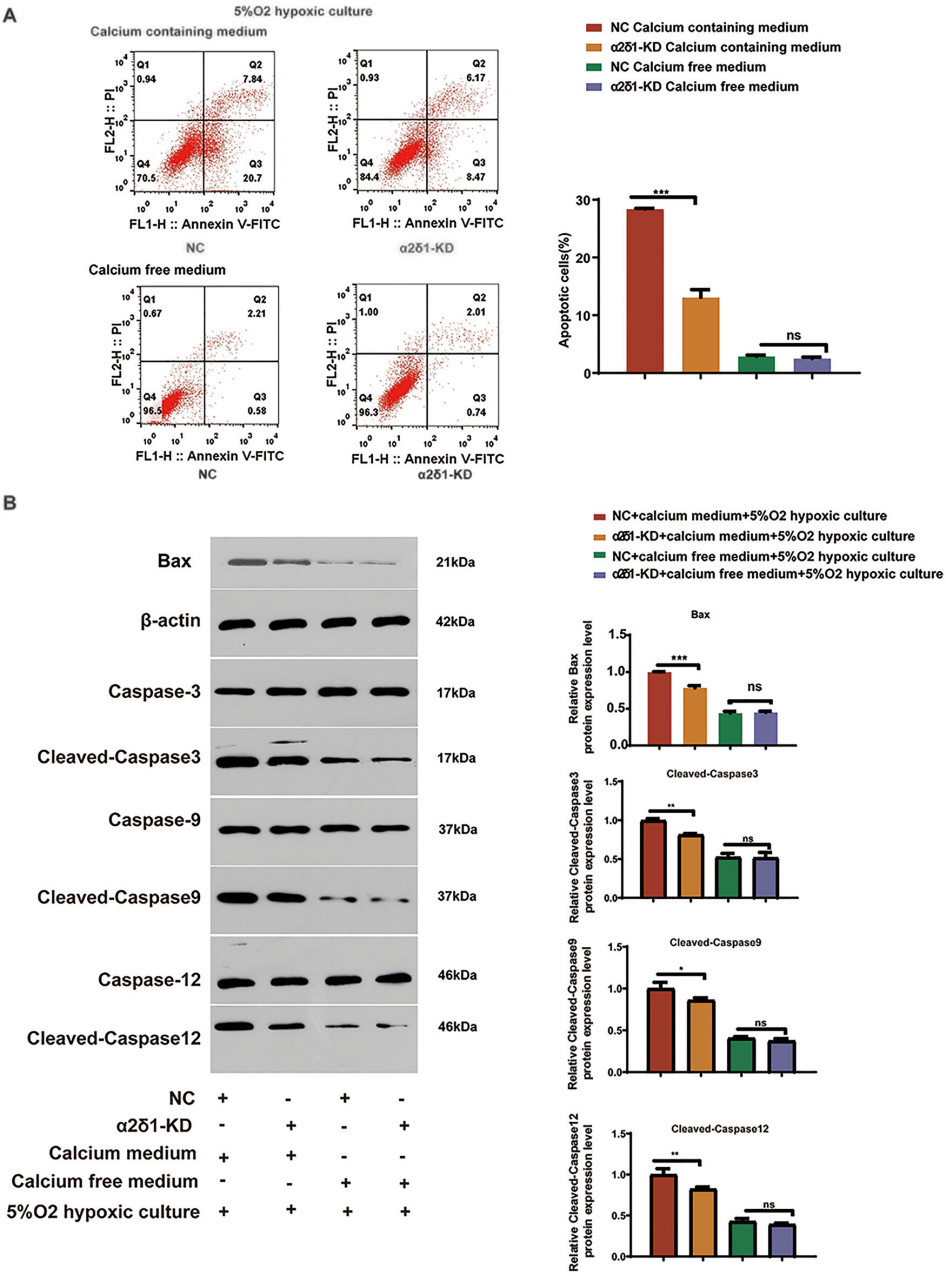
α2δ‐1‐mediated calcium overload promoted BV2 microglia apoptosis. (A) Flow cytometry analysis of BV2 microglia stained with annexin V‐FITC/PI to assess the apoptotic rate in each group. The histogram represents the apoptotic rate. The results demonstrate that cytoplasmic calcium overload promotes BV2 microglia apoptosis by inducing endoplasmic reticulum stress. (B) Western blot analysis of apoptosis‐related proteins in BV2 microglia, including cleaved‐caspase‐3, caspase‐9, caspase‐12, and Bax. β‐actin was used as a loading control. Representative blots from four independent experiments are shown. The results reveal that endoplasmic reticulum stress induced by intracellular calcium overload promotes the expression of apoptosis‐related proteins Cleaved‐caspase‐3, cleaved‐caspase‐9, cleaved‐caspase‐12, and bax. Data are presented as the mean ± standard error of the mean (SEM). *, *P* < 0.05, **, *P* < 0.01, ***, *P* < 0.001. Error bars represent the SEM. Statistical analysis was performed using an unpaired, two‐tailed student's t‐test.

To investigate the effect of endoplasmic reticulum stress (ERS) on BV2 microglia apoptosis, flow cytometry analysis and Western blot were conducted. The results demonstrated that knocking down α2δ‐1 significantly inhibited BV2 microglia apoptosis in the presence of calcium, while no significant change was observed in the absence of calcium (Figure 4A). Western blot analysis further revealed that α2δ‐1 knockdown led to a significant decrease in the protein expression levels of apoptosis‐related proteins, including Cleaved‐Caspase‐3, Cleaved‐Caspase‐9, Cleaved‐Caspase‐12, and Bax, specifically in the calcium‐containing culture (Fig 4B).

## 4 Discussion

Our study investigated the role of α2δ‐1 calcium channels in cerebral hemorrhage and its impact on BV2 microglia. BV2 murine microglial cell lines are extensively utilized in neuroscience research as an immortalized cell line model to study neurodegenerative diseases and related cellular conditions and processes, such as neuroinflammation (Lei et al. 2023). Furthermore, BV2 cells are considered an alternative model system to primary microglial cells. As innate immune sentinels within the central nervous system, microglia sense acute injury‐induced changes rapidly in the microenvironment (Medina et al. 2024). As confirmed by double immunohistochemistry and Western blot analysis, we observed a significant upregulation of α2δ‐1 in cerebral hemorrhage tissue compared to peripheral tissues in 16 patients diagnosed with spontaneous basal ganglia hemorrhage. Notably, our investigation involving microglia, the resident immune cells of the central nervous system (CNS), demonstrated that the heightened expression of α2δ‐1 subsequent to intracerebral hemorrhage plays a pivotal role in facilitating the entry of extracellular calcium ions into the cells. This influx, in turn, triggers PLC‐γ phosphorylation and initiates a series of reactions leading to endoplasmic reticulum stress. Ultimately, these molecular events induce apoptosis in brain cells, contributing to the pathogenesis of secondary brain injury post intracerebral hemorrhage.

The calcium channel α2δ‐1 (Cavα2δ‐1) is an essential functional subunit of voltage‐gated calcium channels (VGCCs) and has been implicated in modulating phosphorylated N‐methyl‐D‐aspartate receptors (NMDAR) activity, promoting their cell surface expression and synaptic function, while also alleviating the Mg2+ block to enhance Ca2+ influx (Wu et al. 2023, Luo et al. 2018). However, the relationship between these two processes, NMDAR activity and calcium overload, is complex and potentially bidirectional. On one hand, α2δ‐1 upregulation could enhance NMDAR signaling, thereby increasing intracellular calcium levels. On the other hand, sustained calcium influx through α2δ‐1 subunit‐containing calcium channels could exacerbate ischemic injury by intensifying oxidative stress and mitochondrial dysfunction, which in turn could further alter NMDAR function. CaVα2δ‐1 protein acts as an auxiliary subunit of VGCCs, regulating the electrokinetic properties of calcium channels and enhancing the expression of calcium ion channels in the cell membrane. It plays a key role in calcium regulation in sensory neurons (Cassidy et al. 2014), and promotes transmitter release in presynaptic axon terminals (Patel et al. 2013). Elevated expression of the CaVα2δ‐1 protein intensifies the current density of calcium channels and boosts glutamate release, contributing significantly to neuronal necrosis following cerebral ischemia (Yoon et al. 2011). R‐phenibut and pregabalin, acting as ligands for the α2δ subunit of these channels, have illustrated neuroprotective capabilities in ischemic rat models offering a promising therapeutic avenue (Lee et al. 2021, Vavers et al. 2016).

Intracellular calcium dysregulation leads to increased phosphorylation of PLC‐γ and generation of IP3. Phospholipase C (PLC), a widely present enzyme in mammalian cells, can be activated by various extracellular factors, such as cell surface antigens, immunoglobulins, cytokines, and growth factors, thereby modulating cell metabolism (Shen et al. 2019). PLC has two subclasses, PLC‐γ1 and PLC‐γ2, which are normally present in the cytoplasm but translocate to the cell membrane upon activation (Wing et al. 2003). Upon activation, PLC hydrolyzes phosphatidylinositol 4, 5‐bisphosphate (PIP2), resulting in the generation of two intracellular products: inositol 3‐phosphate (IP3) and diacylglycerol (DAG). IP3 induces the release of calcium ions from intracellular calcium stores by binding to IP3 receptors (IP3R) present in the endoplasmic reticulum, thereby regulating intracellular calcium signaling pathways (Bill and Vines 2020, Leal et al. 2014) DAG, on the other hand, activates protein kinase C (PKC), triggering a cascade of biochemical reactions involved in cell growth, differentiation, and apoptosis (Shen et al. 2019). Our study demonstrated that intracellular calcium overload promotes the phosphorylation of PLC‐γ and IP3R in BV2 microglia, suggesting a role for α2δ1 in regulating calcium homeostasis.

Under physiological conditions, calcium ions (Ca^2+^) are transported into the endoplasmic reticulum from the cytoplasm through the sarco/endoplasmic reticulum calcium ATPase (SERCA) pumps and released into the cytoplasm via the ryanodine receptors (RyR) and IP3 receptors (Shen et al. 2002). However, in the context of endoplasmic reticulum stress (ERS), the depletion of calcium within the endoplasmic reticulum is the primary cause of apoptosis, rather than an elevation of intracellular Ca^2+^ or an influx of extracellular Ca^2+30^. During the ERS response, calcium pumps within the cytoplasm is activated by increased cytoplasmic Ca^2+^, allowing Ca^2+^ to be transported back into the endoplasmic reticulum (Shen et al. 2019). Additionally, calcium‐binding proteins bind to IP3R and RyR channels, resulting in the release of more Ca^2+^ into the cytoplasm. The elevated cytoplasmic Ca^2+^ concentration activates various Ca^2+^‐dependent degradation enzymes, induces phospholipid breakdown, and damages cell and organelle membranes. Perturbations in endoplasmic reticulum calcium levels, such as the inhibition of ATP‐dependent calcium pumps by toxicocarotene (TG), can trigger calcium release from the endoplasmic reticulum. If extracellular Ca^2+^ replenishment is inadequate, intracellular calcium depletion occurs, leading to ERS‐mediated cell apoptosis (Rao et al. 2002, Zuo et al. 2018) In our study, we also observed a decrease in the expression of ERS‐related proteins following α2δ‐1 gene deletion, suggesting that α2δ‐1 may influence microglial ERS by regulating Ca^2+^ influx after intracerebral hemorrhage, thereby contributing to cell apoptosis.

In the acute stage of intracerebral hemorrhage, clinicians should actively address the increase in intracellular Ca^2+^ concentration and overload, aiming to protect the brain cells surrounding the hematoma. Strategies aimed at improving intracellular Ca^2+^ homeostasis and preventing Ca^2+^ overload could potentially mitigate the detrimental effects of intracellular Ca^2+^ dysregulation and promote neuronal survival.

Despite the significant findings of our study, several limitations should be acknowledged. Firstly, Microglia exhibit diverse responses in disease, varying by spatial, temporal, and functional. Our subsequent studies will examine the role of α2δ proteins in microglial activation and polarization post‐intracerebral hemorrhage (Masuda et al. 2020, Lan et al. 2017). Secondly, our study primarily examined the effects of α2δ‐1 knockdown on BV2 microglia. Future studies may consider integrating R‐phenibut and pregabalin targeting α2δ‐1 to further elucidate its functional role and therapeutic potential in cerebral hemorrhage. Lastly, the clinical implications and translation of our findings require extensive research and clinical trials.

## 5 Conclusion

In conclusion, our study highlights the elevated expression of α2δ‐1 in cerebral hemorrhage tissue and its potential involvement in calcium overload, ER stress induction, and microglial apoptosis. These findings may provide a basis for future studies exploring α2δ‐1 as a potential therapeutic target in intracerebral hemorrhage and secondary brain injury associated with calcium dysregulation. Strategies aimed at improving intracellular Ca^2+^ homeostasis and preventing Ca^2+^ overload could potentially mitigate the detrimental effects of intracellular Ca^2+^ dysregulation.

## Author Contributions

**Ning Yu**: conceptualization, investigation, writing–original draft, methodology, validation, formal analysis, data curation. **Xiaopeng Li**: investigation, writing–review and editing, validation, software, formal analysis, data curation, resources. **Bingqian Wang**: investigation, validation, writing–review and editing, software, formal analysis, data curation, resources. **Chengrui Nan**: investigation, validation, writing–review and editing, software, formal analysis, data curation, resources. **Qianxu Jin**: investigation, validation, writing–review and editing, software, formal analysis, data curation, resources. **Liang Yang**: investigation, validation, writing–review and editing, software, formal analysis, data curation, resources. **Depei Li**: investigation, validation, writing–review and editing, software, formal analysis, data curation, resources. **Zongmao Zhao**: validation, writing–review and editing, conceptualization, project administration, supervision.

### Ethics Statement

This study was conducted in accordance with the Helsinki Declaration and was approved by the Research Ethics Committee of the Second Hospital of Hebei Medical University (Approval letter NO.2017‐R232). A full and detailed consent from the patient/guardian has been taken. The patient's identity has been adequately anonymized. If anything related to the patient's identity is shown, adequate consent has been taken from the patient/relative/guardian.

### Peer Review

The peer review history for this article is available at https://publons.com/publon/10.1002/brb3.70499

## Data Availability

The datasets used and/or analyzed during the current study available from the corresponding author upon reasonable request.
